# Photosynthetic Response of Soybean Leaf to Wide Light-Fluctuation in Maize-Soybean Intercropping System

**DOI:** 10.3389/fpls.2017.01695

**Published:** 2017-09-28

**Authors:** Xingdong Yao, Hongli Zhou, Qian Zhu, Chunhong Li, Huijun Zhang, Jun-Jiang Wu, Futi Xie

**Affiliations:** ^1^Soybean Research Institute, Shenyang Agricultural University, Shenyang, China; ^2^Key Laboratory of Soybean Cultivation of Ministry of Agriculture, Soybean Research Institute, Heilongjiang Academy of Agricultural Sciences, Harbin, China

**Keywords:** high radiation, stress responses, photosynthesis, PSII, photo-inhibition

## Abstract

In maize-soybean intercropping system, soybean plants will be affected by the wide light-fluctuation, which resulted from the shading by maize plants, as the shading of maize the light is not enough for soybean in the early morning and late afternoon, but at noon, the light is strong as the maize shading disappeared. The objective of this study is to evaluate the photosynthetic response of soybean leaf to the wide light-fluctuation. The data of diurnal variation of photosynthetic characters showed that the photosynthetic rate of intercropped soybean was weaker than that of monocropped soybean. The chlorophyll content, ratio of chlorophyll a/b, and AQE (apparent quantum efficiency) were increased and *R*_d_ (dark respiration rate) was decreased for the more efficient interception and absorption of light and carbon gain in intercropping. δ_Ro_ (The efficiency/probability with which an electron from the intersystem electron carriers was transferred to reduce end electron acceptors at the PSI acceptor side) and φ_Ro_ (the quantum yield for the reduction of the end electron acceptors at the PSI acceptor side) in intercropped soybean leaf were lower compared to those in monocropped one, which showed that the acceptor side of PSI might be inhibited, and also it was the main reason that soybean plants showed a low photosynthetic capacity in intercropping. ψ_Eo_ (the efficiency/probability with an electron moves further than Q_A_^-^) in monocropping and intercropping decreased 5.8, and 35.7%, respectively, while φ_Eo_ (quantum yield for electron transport) decreased 27.7 and 45.3% under the high radiation at noon, which suggested that the acceptor side of PSII was inhibited, while the NPQ became higher. These were beneficial to dissipate excess excitation energy in time, and protect the photosynthetic apparatus against photo-damage. The higher performance index on the absorption basis (PI_ABS_) and lower δ_Ro_, φ_Ro_, ψ_Eo_, and φ_Eo_ of intercropped soybeans compared to monocropping under high radiation indicated that the electron transfer of intercropped soybean was inhibited more seriously and intercropped soybean adjusted the electron transport between PSII to PSI to adapt the light-fluctuation. Higher NPQ capacity of intercropped soybeans played a key role in keeping the leaf with a better physiological flexibility under the high radiation.

## Introduction

Light is one of the most important factors affecting plants growth and development (Li A. et al., 2016), with changes in irradiance having impacts on plant growth, morphology, physiology, etc. Maize-soybean intercropping is one of major planting patterns in China, and has contributed significantly to soybean production and to maintain the yield of maize (Yang et al., 2008; Yan et al., 2010; Li et al., 2014). In this intercropping, soybean grow in the rows between maize, and the light situation of soybean canopy is changed by maize (Awal et al., 2006; Yang et al., 2014). The light environment of soybean survived is very complicated. The soybean is shaded by maize at early morning and late afternoon, and exposed to high radiation that higher than light saturation point (LSP) at midday in intercropping (Gong et al., 2015).

The effect of shade on soybean was extensively investigated. In general, plant leaf grown in shade condition was thinner, had a lower net CO_2_ assimilation rate (An) (Tateno and Taneda, 2007), CO_2_ assimilation rate saturated at lower photosynthetic photon flux density (Zhang et al., 2004), and lower amounts of electron transfer carriers than those in unshade condition (Jiang et al., 2011). However, soybean plants grown in intercropping were not only affected by shade, but also affected by high radiation. In this study little was known about the effect of high radiation stress on soybean leaf in intercropping.

High radiation is one of the most frequently stresses that was encountered by plant during growth period. Under high radiation condition, the light energy absorbed by the plant leaf often exceeded the energy required to fix the CO_2_. If the excess excitation energy could not be dissipated in time, it resulted in energy overflow and excessive reactive oxygen species (Foyer and Noctor, 2005; Li et al., 2013; Ruban, 2012). This could be destructive to photosynthetic apparatus. Plants had several regulatory mechanisms to adjust a well-balanced performance of PSI and PSII, and protect photosynthetic apparatus against high radiation (Kono and Terashima, 2014; Kromdijk et al., 2016; Mishanin et al., 2016). Down-regulation of PSII performance is one of the most efficient mechanisms of photoprotection (Müller et al., 2001; Mishanin et al., 2016). This mechanism decreased in the quantum yield of PSII, the capacity of photosynthetic electron transport and photochemical quenching, while increased in NPQ, which provided enhanced dissipation of energy in the light-harvesting complex (Ruban et al., 2012; Niyogi and Truong, 2013; Mishanin et al., 2016). It is significant that plant dissipate excess solar radiation through NPQ to maintain optimal rates of photosynthesis and provide the plant against oxidative damage (Mishanin et al., 2016).

The leaf of intercropped soybean was exposed to high radiation for several hours at midday. However, little was known about the acclimation of soybean plants grown in intercropping to high radiation. And more effort should be done to study the mechanisms of photoprotection of PSI and PSII to strong fluctuations of environment light (Allakhverdiev and Murata, 2004; Allahverdiyeva et al., 2014). Chlorophyll a fluorescence is an important method for studying PSII function and reaction under different environmental conditions (Allakhverdiev and Murata, 2004; Strasser et al., 2004; Kalaji et al., 2017), and it can be used to analyze the changes of reaction center, the efficiency of electron transfer from PSII to the acceptor side of PSI in the intersystem chain under different growth conditions (Tóth et al., 2007; Tsimilli-Michael and Strasser, 2008; Strasser et al., 2010; Kalaji et al., 2017). Therefore, chlorophyll a fluorescence is used to study the effect of fluctuation light on plant.

In this study, the diurnal variation of photosynthesis characteristics, fast and slow chlorophyll fluorescence, morphological characteristic of soybean leaf grown in intercropping and monocropping were measured to understand light acclimation of soybean grown under different planting pattern. The objective of this study is to evaluate the photosynthetic response of soybean leaf to the wide light-fluctuation in intercropping. This study provides insights into the physiological flexibility of soybean adapt to light-fluctuation in intercropping.

## Materials and Methods

### Plant Material and Experimental Design

Field experiments was carried out from May 2015 to October 2015 at the experimental farm of Shenyang Agricultural University, Shenyang, Liaoning Province, China, The experiment was laid out a completely randomized block design with two cropping (maize-soybean intercropping and soybean monocropping). The row direction was north–south layout. Soybean and maize were sown on May 3rd, 2015. Soybean cultivar Liaodou32 was used, and maize cultivar used was Zhengdan958. The intercropping used wide-narrow row planting, and the ratio of maize to soybean rows in the intercropping was 2:2. The distance between the maize and soybean was 80 cm, and the distance between two rows of maize or two rows of soybean was 40 cm. The densities of sole cropping soybean, intercropped soybean and intercropped maize were 150000, 150000, and 60000 plants ha^-1^. The uppermost and fully expanded leaves were used for measurements at R2 stage (full flowering).

### Determination of Light Conditions

**FIGURE 1:**
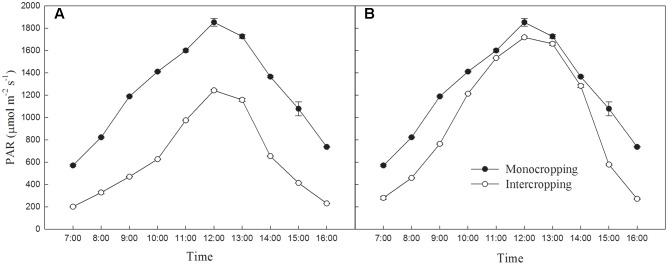
**(A)** Diurnal variation of average PAR on the soybean canopy during July 25th, 2015 in Shenyang. **(B)** Diurnal variation of maximum PAR on the soybean leaf during July 25th, 2015 in Shenyang.

The average PAR and maximum PAR of soybean canopy changes of different cropping were measured in a sunny day using a light meter (AccuPAR LP-80, United States) according to the method of Yang et al. (2014), and listed on **Figure 1**.

### Photosynthetic Parameters

Light response curves of Photosynthesis were measured using a LI-6400XT (Li-Cor, United States). The parameters were measured on uppermost and fully expanded leaves from 09:00 to 11:30 am on a clear day. The temperature and CO_2_ concentration of leaf chamber were maintained at 25°C and 380 μmol mol^-1^, respectively. PAR was increased from 0 to 1500 μmol photons m^-2^ s^-1^ (0, 20, 50, 80, 100, 200, 400, 600, 800, 1000, 1200, 1500 μmol m^-2^ s^-1^, 36 min). And then, after linear fitting, light compensation point (LCP), LSP and light-saturated net photosynthetic rate (*A*_max_), apparent quantum efficiency (AQE) and dark respiration rate (*R*_d_) were estimated by the method of Ye (2007).

Diurnal variation of leaf gas exchange was measured on a clear sunny day. Photosynthesis was measured with a LI-6400XT (Li-Cor, United States) equipped with 2 cm × 3 cm clear chamber. *P*_n_ and *C*_i_ were recorded at intervals of 2 h from 08:30 am to 16:30 pm. The measured leaves were kept at their natural angle of posture exposing to direct irradiance outside leaf chamber. The temperature and CO_2_ concentration of leaf chamber were kept at natural environment.

### Chlorophyll Fluorescence

Light response curves for fluorescence were monitored by PAM-2500 chlorophyll fluorometer (Heinz Walz GmbH, Germany), and according to the method of Chen et al. (2014). Rapid light curves were performed with gradually increasing irradiance in 11 steps with 180 s intervals. For each step, the irradiance is 0, 198, 363, 619, 785, 981, 1160, 1386, and 1663 μmol m^-2^ s^-1^, and the fluorescence signal was recorded, respectively. The data were recorded and read data from the PamWin V3.12g (system control and data acquisition system).

Diurnal variation of leaf chlorophyll fluorescence was measured on a clear sunny day by PAM-2500 chlorophyll fluorometer (Heinz Walz GmbH, Germany). The fluorescence signals were recorded at intervals of 2 h from 08:30 am to 16:30 pm. The measured leaves were kept at their natural angle of posture exposing to direct irradiance outside leaf chamber. Then, the Y(II) and other parameters were calculated as described by Baker (2008).

### Chlorophyll a Fluorescence Transient

After a dark adaptation for 30 min, chlorophyll a fluorescence transient (OJIP) of soybean leaves were measured by the plant efficiency analyzer (Hansatech Instruments Ltd., Norfolk, United Kingdom) in a solar day at 10:00 am to 12:30 pm. The uppermost and fully expanded leaves were used for measurements. We obtained the parameters of chlorophyll a fluorescence which could reflect the PSII activity of soybean leaves. Then, the PSII parameters derived from the OJIP transient were analyzed based on the method of Strasser et al. (2004, 2010).

### Leaf Chlorophyll Content, Morphological and Anatomical Features

After the measurements described above completed, the leaves were collected for determination of chlorophyll content (Chl a, Chl b, Chl a+b, Chl a/b). Chlorophyll pigments were extracted by grinding leaves in 80% acetone in the dark at room temperature and were expressed as mg/g FW from the equations of Porra (2002). The leaf area was measured by a portable leaf area meter (LI-3100C, LI- COR, United States).

The middle segments of the uppermost and fully expanded leaves were sampled and fixed in a formaldehyde solution (FAA). Leaf segments were dehydrated, cleared and embedded in paraffin. Then these samples were cut by RM2235 rotary microtome (Leica Microsystems Ltd., Germany) at thickness of 10 μm. Sections were stained with Safranin O and Fast green, then observed and captured by Axio Imager A2 microscope (Zeiss, Germany). Leaf thickness, palisade tissue thickness and spongy tissue thickness were quantified by using ZEN imaging software (Zeiss, Germany).

### Determination of Malondialdehyde (MDA) Content and Activity of Antioxidant Enzymes

The middle segments of the uppermost and fully expanded leaves were collected at 12:30, and immediately stored in liquid nitrogen, and then kept at -80°C. Leaf sample was homogenized with 50 mM phosphate buffer (pH 7.8) containing 10 mM Polyvinylpyrrolidone (PVP) and 0.2 mM EDTA in an ice bath, and centrifuged at 12,000 × *g* and 4°C for 20 min. The supernatant was used for MDA and enzyme analysis. The MDA content was assayed by the thiobarbituric acid test (Hodges et al., 1999). Activity of antioxidant enzymes was measured according to Samantary (2002). The activity of superoxide dismutase (SOD) was assayed by measuring its ability to inhibit the photochemical reduction of NBT at 560 nm, and was expressed as units per g of fresh weight. The activity of catalase (CAT) was determined by measuring the decrease of oxidized phenols of H_2_O_2_ at 240 nm, and the activity of CAT was expressed as units per g of fresh weight during 1 min.

### Data Analysis

The experiments were arranged in a completely randomized block design with three replications. One-way analysis of variance (ANOVA) and the Duncan’s multiple range tests were used to assess each of the parameters using SPSS statistics software (Version 20, SPSS, Chicago, IL, United States). The graphs were made using Sigmaplot (Version 12, Systat Software).

## Results

### Effect of Different Planting Pattern on PAR of Soybean Population

The light environment of different planting patterns was showed in **Figure 1**. The average PAR on the soybean canopy in intercropping was significantly lower than those in monocropping. The maximum PAR on the soybean leaf was significantly lower than those in monocropping in early morning and late afternoon, but was exposed to high radiation at noon.

### Effect of Different Planting Pattern on Chlorophyll Content, Morphology of Soybean Leaf and Light Response Curve of Photosynthesis

Leaf in intercropping showed a significantly higher photosynthetic pigment concentration per fresh weight, and significantly lower chla/b than those under monocropping (**Table 1**). The leaf area per plant in intercropping was significantly lower than that in monocropping.

**Table 1:** The content of chlorophylls, leaf area and morphological characteristic of soybean leaves under monocropping and intercropping.

Treatment	Content (mg/g)			Chl a/b	LA (m^2^)	LT (μm)	PTT (μm)	STT (μm)	PTT/STT
	Chl a	Chl b	Chl a+b						
Monocropping	2.97 ± 0.04^b^	0.82 ± 0.01^b^	3.79 ± 0.05^b^	3.62 ± 0.02^a^	0.25 ± 0.06^a^	131.8 ± 3.4^a^	55.9 ± 3.3^a^	54.5 ± 3.1^a^	1.03^a^
Intercropping	3.41 ± 0.05^a^	1.03 ± 0.01^a^	4.44 ± 0.06^a^	3.31 ± 0.02^b^	0.18 ± 0.09^b^	107.2 ± 1.6^b^	31.8 ± 1.6^b^	53.2 ± 1.6^a^	0.60^b^

*Mean values ± SE from five replicates, and different letters indicate statistical difference significance at *P* < 0.05 among the treatments by Duncan’s multiple range tests. Chl, chlorophyll; LA, leaf area; LT, leaf thickness; PTT, palisade tissue thickness; STT, spongy tissue thickness*.

In contrast to soybean grown in monocropping, the leaf became thinner, and the thickness of both leaf and palisade tissue were significantly decreased, however, the spongy tissue thickness was little changed.

**FIGURE 2:**
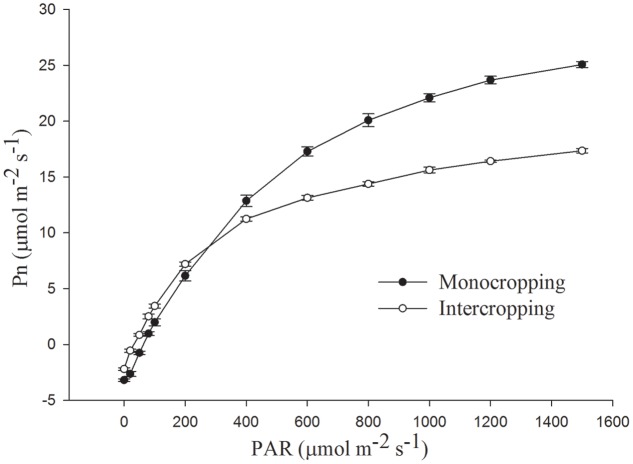
Net photosynthetic rate, measured as CO_2_ uptake in soybean leaf under monocropping and intercropping.

*P*_n_ increased rapidly as PAR increased to 600 μmol⋅m^-2^⋅s^-1^ and then increased slowly to saturation (**Figure 2**). *P*_n_ under intercropping was higher than that in monocropping at low PAR, while lower at high PAR. *A*_max_ (light-saturated net photosynthetic rate) of soybean leaf in intercropping was about 18.96 μmol⋅m^-2^⋅s^-1^, it was only about 65.79% of *A*_max_ in monocropping (28.82 μmol⋅m^-2^⋅s^-1^, **Table 2**). The LCP, LSP, and *R*_d_ (dark respiration rate) in intercropping were lower than those in monocropping, while AQE was higher than those in monocropping.

**Table 2:** Effect of shade on the photosynthetic parameters of soybean leaves.

Treatment	*A*_max_ (mol m^-2^ s^-1^)	LCP (mol m^-2^ s^-1^)	LSP (mol m^-2^ s^-1^)	AQE (mol m^-2^ s^-1^)	*R*_d_ (mol m^-2^ s^-1^)
Monocropping	28.82 ± 0.93^a^	60 ± 2.2^a^	1671 ± 35^a^	0.053 ± 0.003^b^	-3.19 ± 0.81^b^
Intercropping	18.96 ± 1.12^b^	36 ± 1.6^b^	1176 ± 24^b^	0.061 ± 0.005^a^	-2.20 ± 0.74^a^

*Mean values ± SE from five replicates, and different letters indicate statistical difference significance at *P* < 0.05 among the treatments by Duncan’s multiple range tests. *A*_*max*_, light-saturated net photosynthetic rate; LCP, light compensation point; LSP, light saturation point; AQE, apparent quantum efficiency; *R*_*d*_, dark respiration rate*.

### Effect of Different Planting Pattern on Rapid Light Response Curve of Soybean Leaf

**FIGURE 3:**
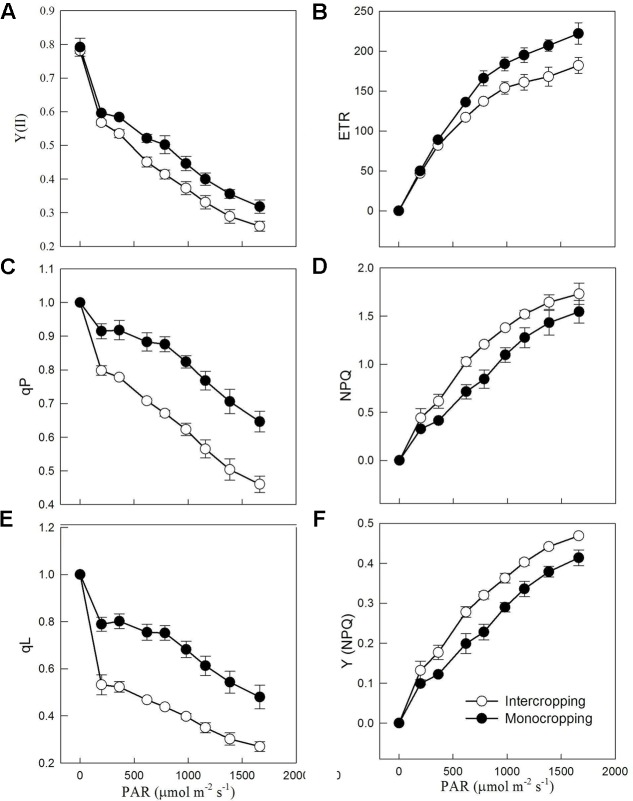
Chlorophyll a fluorescence parameters derived from the rapid light curves in monocropping and intercropping. **(A)** Y(II), the photochemical efficiency of PSII, **(B)** ETR, electron transport rate, **(C)** qP, coefficients estimating the fraction of open PS II reaction centers based on a puddle model, **(D)** NPQ, non-photochemical quenching, **(E)** qL, coefficients estimating the fraction of open PS II reaction centers based on a lake model, and **(F)** Y(NPQ), quantum yield of non-photochemical quenching.

Results obtained from rapid light curves showed that Y(II) (quantum yield of photochemical energy conversion in PS II), qP (coefficients estimating the fraction of open PS II reaction centers based on a puddle model), and qL (coefficients estimating the fraction of open PS II reaction centers based on a lake model) were decreased gradually with the increase of PAR (**Figures 3A,C,E**). And Y(II), qP and qL in intercropping were higher than those in monocropping. ETR (electron transport rate) increased significantly with the increase of PAR, and ETR in intercropping saturated at lower PAR than those in monocropping (**Figure 3B**). NPQ (non-photochemical quenching) and Y(NPQ), expressed the thermal dissipation of excitation energy, had a significant rise with the increase of PAR (**Figures 3D,F**).

### Diurnal Variation of Leaf Gas Exchange and Chlorophyll a Fluorescence

**FIGURE 4:**
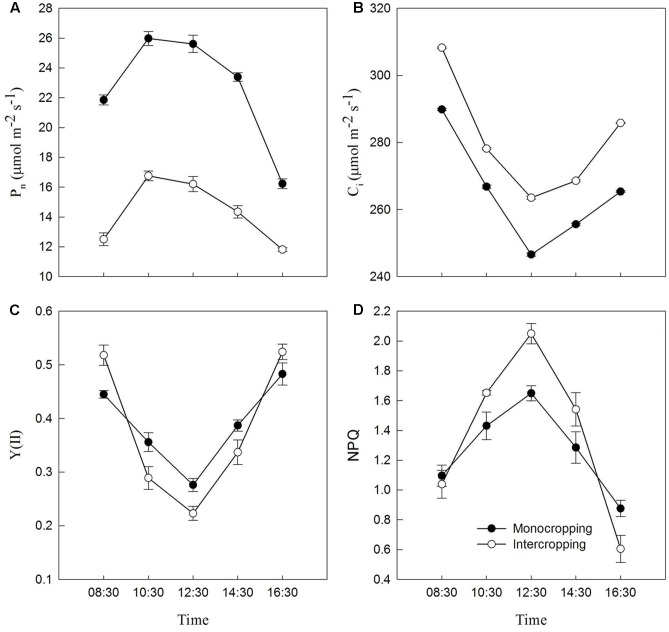
Diurnal variation of *P*_n_, *C*_i_, Y(II), and NPQ of soybean leaf in monocropping and intercropping. **(A)** Pn, net photosynthetic rate, **(B)** Ci, intercellular CO_2_ concentration, **(C)** Y(II), the photochemical efficiency of PSII, and **(D)** NPQ, non-photochemical quenching parameter.

*P*_n_ increased with the increase of light intensity, and reached maximum at 10:30, and then began to decrease. *P*_n_ in intercropping was significantly lower than that in monocropping (**Figure 4A**). *C*_i_ and Y(II) decreased with the increase of light intensity, and reached minimum at noon, and then began to recover. *C*_i_ in intercropping was significantly higher than that in monocropping. Y(II) in intercropping was significantly lower than that in monocropping at 10:30–14:30 (**Figures 4B,C**). NPQ increased with the increasing of light intensity, and reached maximum at noon, then began to decrease. And NPQ in intercropping was significantly higher than that in monocropping at 10:30–14:30 (**Figure 4D**).

### Effect of High Radiation on Slow Kinetics of Chlorophyll a Fluorescence Induction at Noon

At noon, the NPQ (non-photochemical quenching) and qN (coefficients of non-photochemical quenching) in intercropping were significantly higher than those in monocropping, while qP (coefficients estimating the fraction of open PS II centers based on a puddle model) and qL (coefficients estimating the fraction of open PS II centers based on a lake model) in intercropping was significantly lower than those in monocropping (**Table 3**).

**Table 3:** Effect of high light on the mode of the yields for dissipative processes for the energy absorbed by PSII of soybean at midday (12:30 pm).

Treatment	NPQ	qN	qP	qL
Monocropping	1.649 ± 0.051^b^	0.81 ± 0.022^b^	0.758 ± 0.024^a^	0.643 ± 0.015^a^
Intercropping	2.049 ± 0.068^a^	0.866 ± 0.006^a^	0.629 ± 0.014^b^	0.525 ± 0.007^b^

*Mean values ± SE from five replicates, and different letters indicate statistical difference significance at *P* < 0.05 among the treatments by Duncan’s multiple range tests. NPQ, non-photochemical quenching parameter; qN, coefficients of non-photochemical quenching; qP, coefficients estimating the fraction of open PS II reaction centers based on a puddle model; qL, coefficients estimating the fraction of open PS II reaction centers based on a lake model*.

At noon, Y(II) in intercropping was significantly lower than those in monocropping, while Y(NPQ) in intercropping was significantly higher than those in monocropping. Y(NO) in intercropping was lower than those in monocropping, but there was no significant difference between them (**Table 4**).

**Table 4:** Effect of high light on the mode of the yields for dissipative processes for the energy absorbed by PSII of soybean at midday (12:30 pm).

Treatment	Y(II)	Y(NPQ)	Y(NO)
Monocropping	0.276 ± 0.012^a^	0.463 ± 0.010^b^	0.261 ± 0.003^a^
Intercropping	0.223 ± 0.013^b^	0.531 ± 0.018^a^	0.253 ± 0.005^a^

*Mean values ± SE from five replicates, and different letters indicate statistical difference significance at *P* < 0.05 among the treatments by Duncan’s multiple range tests. The sum of Y(II), Y(NPQ), and Y(NO) is unity. Y(II), quantum yield of photochemical energy conversion in PS II; Y(NPQ), the quantum yield of regulated non-photochemical energy loss in PS II; Y(NO), quantum yield of non-regulated non-photochemical energy loss in PS II*.

### Effect of High Radiation on Fast Chlorophyll Fluorescence Kinetic in Monocropping and Intercropping

The fluorescence parameters derived from fast fluorescence kinetic are listed in **Table 5**. At 10:00 am, φ_Po_ (maximal quantum yield of primary photochemistry), ψ_Eo_ (efficiency/probability that an electron moves further than Q_A_^-^), φ_Eo_ (quantum yield for electron transport), PI_ABS_ (performance index on the absorption basis) and *W*_k_ (the ratio of variable fluorescence at the K-step to the fluorescence difference *F*_j_-*F*_o)_ in intercropping were significantly higher than those in monocropping, while δ_Ro_ (efficiency/probability with which an electron from the intersystem electron carriers is transferred to reduce end electron acceptors at the PSI acceptor side) in intercropping was significantly lower than those in monocropping. At midday (12:30 pm), ψ_Eo_, φ_Eo_, δ_Ro_, and φ_Ro_ in intercropping became lower than those in monocropping, while *W*_k_ in intercropping were higher than those in monocropping.

**Table 5:** Selected parameters derived from fast fluorescence kinetic measurements in soybean leaves at 10:00 am and 12:30 pm (the PAR of soybean leaf under intercropping and monocropping were 1213 and 1411 μmol m^-2^ s^-1^ at 10:00 am, while the PAR of soybean leaf under intercropping and monocropping were 1750 and 1860 μmol m^-2^ s^-1^ at 12:30 pm).

	Monocropping		Intercropping	
	10:00 am	12:30 pm	10:00 am	12:30 pm
φ_Po_	0.794 ± 0.003^b^	0.635 ± 0.006^d^	0.827 ± 0.010^a^	0.679 ± 0.011^c^
ψ_Eo_	0.605 ± 0.011^b^	0.570 ± 0.014^c^	0.656 ± 0.009^a^	0.422 ± 0.005^d^
φ_Eo_	0.509 ± 0.009^b^	0.368 ± 0.002^c^	0.545 ± 0.002^a^	0.298 ± 0.004^d^
σ_Ro_	0.551 ± 0.020^c^	1.335 ± 0.047^a^	0.505 ± 0.003^d^	1.064 ± 0.038^b^
φ_Ro_	0.276 ± 0.011^c^	0.480 ± 0.003^a^	0.274 ± 0.002^c^	0.322 ± 0.015^b^
PI_abs_	5.282 ± 0.146^b^	0.624 ± 0.016^c^	6.574 ± 0.165^a^	0.638 ± 0.012^c^
*W*_k_	0.324 ± 0.006^d^	0.740 ± 0.013^b^	0.344 ± 0.009^c^	0.832 ± 0.009^a^

*Mean values ± SE from five replicates, and different letters indicate statistical difference significance at *P* < 0.05 among the treatments by Duncan’s multiple range tests. φ_*Po*_, maximal quantum yield of primary photochemistry; ψ_*Eo*_, efficiency/probability that an electron moves further than Q_*A*_^-^; φ_*Eo*_, quantum yield for electron transport; δ_*Ro*_, efficiency/probability with which an electron from the intersystem electron carriers is transferred to reduce end electron acceptors at the PSI acceptor side; φ_*Ro*_, quantum yield for reduction in end electron acceptors at the PSI acceptor side; PI_*ABS*_, performance index on the absorption basis; *W*_*k*_, the ratio of variable fluorescence at the K-step to the fluorescence difference *F*_*j*_-*F*_*o*_*.

### The Lipid Peroxidation and ROS Scavenging Metabolism

The MDA content and activity of antioxidant enzymes were showed in **Table 6**. The MDA content, activities of SOD and CAT in intercropping were significantly higher than those in monocropping at noon.

**Table 6:** The MDA and activity of antioxidant enzymes in intercropping and monocropping at noon.

Treatment	MDA (μmol g^-1^ FW)	SOD (U g^-1^ FW min^-1^)	CAT (U g^-1^ FW min^-1^)
Monocropping	65 ± 2.0^b^	260 ± 2.5^b^	524 ± 8.5^b^
Intercropping	86 ± 2.6^a^	329 ± 3.6^a^	819 ± 5.3^a^

*Mean values ± SE from five replicates, and different letters indicate statistical difference significance at *P* < 0.05 among the treatments by Duncan’s multiple range tests*.

## Discussion

### The Change of Photosynthesis Capacity in Intercropping

In intercropping system, high crop significantly reduced the PAR for soybean, and soybean had to make some response to adapt the change of light environment. The decrease of LCP, LSP, and *A*_max_ (light-saturated net photosynthetic rate) in intercropping indicated that the photosynthetic capacity was limited. The increase of AQE indicated that the ability of light-intercepting gets promoted in light-limited environment conditions, and this was beneficial for higher light utilization efficiency in intercropping. The increase of *R*_d_ (dark respiration rate) indicated that soybean in intercropping dropped the energy expenditure. All these features contributed to the efficient interception and absorption of light and carbon gain in intercropping. And these were similar to that plant grew in shade condition (Zhang et al., 2004; Tateno and Taneda, 2007; Gong et al., 2014). Therefore, the shade of maize leaded to the decrease of photosynthetic capacity of soybean leaf in intercropping. And the shade-tolerant and high photosynthetic efficiency soybean cultivar could be choosed to improve the photosynthetic capacity and yield of soybean in intercropping (Liu et al., 2014; Cui et al., 2015).

The decrease of photosynthetic capacity was caused by stomatal or non-stomatal limitations (Gong et al., 2015). Previous study suggested that the decrease of photosynthetic capacity of spring barley in shade condition was not caused by stomatal effect (Zivcak et al., 2014). Our result showed that *P*_n_ was limited in intercropping, however, *C*_i_ inside the leaf in intercropping was higher than that in monocropping (**Figure 4**). This research showed the same result that the decrease of *P*_n_ in intercropping was not caused by stomatal effect.

Leaf photosynthetic rate is related to chlorophyll content (Shao et al., 2014). Chl a is essential for determining photosynthesis, and Chl b determine the wavelengths of light that can be absorbed by the organism (Field et al., 2013). Intercropped soybean leaf contained more chl a and chl b content per weight and had lower chl a/b than those in monocropping, which could broaden the wavelengths of light that could be absorbed, and effectively increase the ability of light capture (Gong et al., 2014). This is an important adaptation for plants growing in shaded environments.

The leaf and palisade tissue thickness of soybean leaf in intercropping became thinner, which resulted in the reduction of chloroplast, where carboxylation reactions of photosynthesis take place, mostly located in palisade tissue (Terashima et al., 2006; Gong et al., 2015). Therefore, thinner palisade tissue in intercropping decreased the photosynthetic capacity of soybean leaf.

The higher PI_ABS_ (performance index on the absorption basis) and φ_Po_ (maximal quantum yield of primary photochemistry) in intercropping indicated that the light-intercepting capacity and PSII activity was enhanced. But the δ_Ro_ (the efficiency/probability with which an electron from the intersystem electron carrier s was transferred to reduce end electron acceptors at the PSI acceptor side) and φ_Ro_ (the quantum yield for the reduction of the end electron acceptors at the PSI acceptor side) of the plants grown in intercropping were lower than those of the monocropped plants (**Table 5**). And this indicated that the quantum efficiency from PSII to PSI in intercropping were lower than that in monocropping, electron transport between Q_B_ and PSI and the acceptor side of PSI might be inhibited (Wang et al., 2006; Li et al., 2014; Li L. et al., 2016; Zivcak et al., 2014). Intercropped soybean plants increased the photochemical efficiency of PSII, but the electron transport was limited and the accepted capacity of PSI was low. This was one of the reasons that soybean growth was inhibited and showed a low photosynthetic capacity in intercropping. Therefore, the limitation of electron transport and the changing of morphology of soybean leaf in intercropping were the reason that the photosynthetic capacity of soybean cultivars decreased.

### The Acclimation of Soybean Leaf on High Radiation at Noon

As shown in **Figure 1B**, intercropped soybean leaf were exposed to high radiation at noon, and had to take a series of reactions to adapt it. Leaf in intercropping exhibited higher Y(II) and lower NPQ than those in monocropping in the morning and afternoon, indicated the higher efficiency of light utilization at low radiation. However, leaf in intercropping showed lower Y(II) and higher NPQ than those in monocropping at noon (from 10:30 to 14:30), this indicated that the absorbed energy of PSII flux to photochemical processes reduced and this part of energy converted into the non-photochemical energy loss or non-photochemical quenching in high radiation. Higher NPQ indicates a higher transthylakoid proton gradient (ΔpH), which leads to more efficient downregulation of electron transport from PSII to PSI, hence, lower risk of hydroxyl radical production on PSI (Joliot and Johnson, 2011; Brestic et al., 2015). These all were beneficial for dissipating excess excitation energy in time and avoiding photo-damage. The lower qL in intercropping suggested that soybean plants grown in intercropping could close or inactivate more reaction centers to limit the energy input into PSII in high radiation (**Table 3**).

The fate of absorbed light energy was shown in **Table 4**. Y(NPQ) is an important indicator to reflect photoprotection. In high radiation, Y(II) in intercropping reduced, while Y(NPQ) increased significantly. The significant increase of Y(NPQ) suggested more absorbed energy flux from the photochemical energy conversion to the regulated non-photochemical energy loss in PSII in intercropping in order to adapt high radiation condition. Higher Y(NPQ) implied that there was still photochemical energy conversion or protective regulatory mechanisms to dissipate the light energy absorbed by soybean plants. Y(NO) is an important indicator of photo-damage. There is no significant difference in Y(NO) between intercropping and monocropping, which indicated that wide light-fluctuation in intercropping did not cause photo-damage. The high excitation pressure is considered to be directly related to the photo-damage (Kornyeyev et al., 2010; Zivcak et al., 2014), and is easy to happen at high light. Together with low LSP, *A*_max_, and ETR in intercropping, we could expect severe photo-damage in intercropping. However, there were low differences in photo-damage. One possible explanation is that the photo-protection ability is increased to avoid photo-inhibition with the increasing of excitation pressure at high light (Niinemets and Kull, 2001).

The higher *W*_k_ in intercropping demonstrated that the donor side of PSII was seriously inhibited compared to monocropping in high radiation at noon (Chen et al., 2004; Li L. et al., 2016). The higher ψ_Eo_ and φ_Eo_ at 10:30 suggested that the quantum efficiencies in PSII electron transfer chain of soybean plants grown in intercropping were enhanced compared to the monocropped soybean. At 12:30, with the effect of high radiation, the ψ_Eo_ and φ_Eo_ in intercropping and monocropping decreased; the ψ_Eo_ and φ_Eo_ in intercropping were lower than those in monocropping. The higher decrease of parameters ψ_Eo_ and φ_Eo_ in intercropping reflects higher light susceptibility to high radiation. These indicated photo-inhibition of soybean leaf grown in intercropping caused a huge accumulation of Q_A_^-^ (Strasser et al., 2004). Excess electrons transported from PSII to the acceptor side of PSI may result in the occurring of photo-inhibition (Huang et al., 2015). Thus, we expected that soybean leaf grown in intercropping was more susceptible to photo-inhibition in high radiation. However, the lower PSII connectivity of shade leaves might keep the excitation pressure lower, physiologically more acceptable level and thus protected photosynthetic apparatus against high light (Zivcak et al., 2014).

MDA content is used as an indicator of lipid peroxidation (Sudhakar et al., 2001; Spicher et al., 2016). In our study, The MDA content of Intercropped soybean leaf was significantly higher than monocropped one. And this indicated that the higher accumulation of ROS led to much more membrane peroxidation within the thylakoid and chloroplasts in intercropping than this in monocropping. The higher excess excitation energy and the lower electron transportation activity between PSII and PSI in intercropping probably turns the photosynthetic apparatus into a stronger ROS source (Gill and Tuteja, 2010; Vanlerberghe et al., 2016). Antioxidative defense mechanisms can scavenge the ROS to protect the photosynthetic apparatus. In our study, intercropping increased the activities of SOD and CAT in soybean leaf to scavenge the higher production of ROS. And this was beneficial for the photosynthetic apparatus to against oxidative stress. Together with no significant difference in Y(NO) between intercropping and monocropping, these suggested that although there was a higher ROS in intercropping, the higher activity of antioxidant enzymes could scavenge the ROS in time to be not causing photo-damage.

## Conclusion

Soybean leaf had a sufficient physiological flexibility to respond to change of light radiation. The photosynthetic capacity of soybean plants grown in intercropping was limited; and it was associated with the block of electron transport from PSII to PSI. In high radiation, the electron transport from PSII to PSI and NPQ were increased significantly, but acceptor side of PSII was inhibited, this was beneficial to keep the excitation pressure lower and protect the photosynthetic apparatus against photo-damage. Meanwhile, the activity of antioxidant enzymes were increased to against oxidative stress. Soybean leaf in intercropping showed a higher light susceptibility to high radiation and adapted the light-fluctuation by adjusting the electron transport between PSII to PSI.

## Author Contributions

FX and XY conceived and designed research. XY performed the experiments, analyzed the data, wrote the manuscript. FX revised the manuscript. HoZ, and QZ helped in conducting the experiments and analyzing the data. CL, HuZ, and J-JW critically edited the manuscript.

## Conflict of Interest Statement

The authors declare that the research was conducted in the absence of any commercial or financial relationships that could be construed as a potential conflict of interest.
